# A critical developmental window for ELAV/Hu-dependent mRNA signatures at the onset of neuronal differentiation

**DOI:** 10.1016/j.celrep.2022.111542

**Published:** 2022-10-25

**Authors:** Judit Carrasco, Fernando Mateos, Valérie Hilgers

**Affiliations:** 1 Max-Planck-Institute of Immunobiology and Epigenetics, 79108 Freiburg, Germany; 2 Faculty of Biology, Albert Ludwig University, 79104 Freiburg, Germany; 3 International Max Planck Research School for Molecular and Cellular Biology (IMPRS-MCB), 79108 Freiburg, Germany

**Keywords:** nervous system, differentiation, ELAV/Hu proteins, FNE, RNA processing, alternative polyadenylation, alternative splicing, *Drosophila*, exon-activated rescue, EXAR

## Abstract

Cell-type-specific gene regulatory programs are essential for cell differentiation and function. In animal neurons, the highly conserved ELAV/Hu family of proteins promotes alternative splicing and polyadenylation of mRNA precursors to create unique neuronal transcript isoforms. Here, we assess transcriptome profiles and neurogenesis success in *Drosophila* models engineered to express differing levels of ELAV activity in the course of development. We show that the ELAV-mediated establishment of a subset of neuronal mRNA isoforms at the onset of neuron differentiation constitutes a developmental bottleneck that cannot be overcome later by the nuclear activation of the paralog found in neurons (FNE). Loss of ELAV function outside of that critical time window results in neurological defects. We find that FNE, when activated early enough, can restore ELAV-dependent neuronal mRNA isoforms and fully rescue development. Our findings demonstrate the essential role of robust cellular strategies to maintain ELAV activity and intact neuronal signatures in neurogenesis and neuronal function.

## Highlights

• *Drosophila* ELAV establishes alternative mRNAs at the onset of neuronal differentiation
• Loss of early neuronal mRNA isoforms irremediably impairs neurogenesis
• After the critical time window, nuclear activation of FNE rescues ELAV loss *in vivo*
• If activated at differentiation onset, FNE fully restores neuronal RNAs and development

## Graphical abstract

**Figure:**
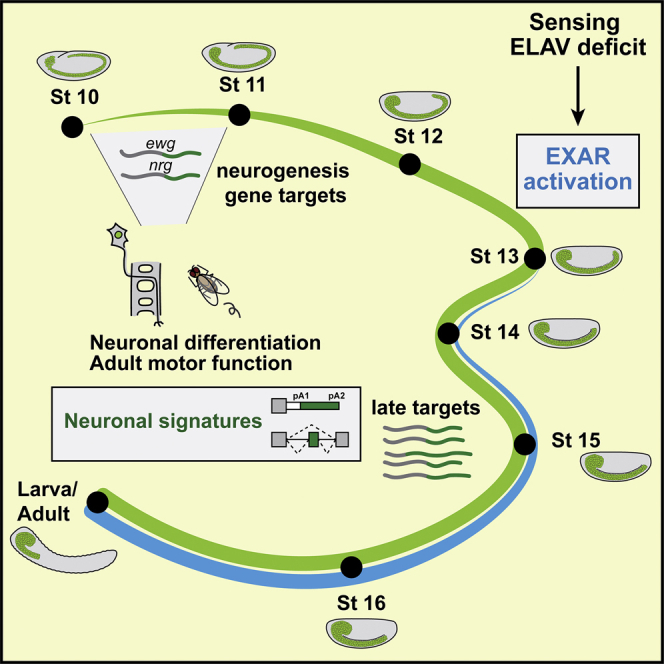

## Teaser

Carrasco et al. show that neuronal differentiation depends on the establishment of a subset of alternative mRNA isoforms by one single effector, ELAV, as soon as the first post-mitotic neurons are born. Outside of this developmental window, nuclear activation of the paralog FNE fully rescues neuronal RNA signatures and neurogenesis.

## Introduction

Cells adopt many strategies to increase the coding and regulatory potential of their genetic content. In the nervous system, a tissue displaying an astonishing transcriptome diversity, the production of multiple mRNA isoforms from a single protein-coding gene is particularly prevalent (Andreassi et al., 2018; Lianoglou et al., 2013). Hundreds of genes undergo neuron-specific processing of mRNA precursors, in the form of differential inclusion of exons through alternative splicing (AS), and the alternative use of transcription end sites through alternative polyadenylation (APA). While in other tissues APA can generate shorter or longer transcripts, neuronal APA is characterized by a consistent shift toward distal sites. Depending on the location of the polyadenylation site, neuronal APA results in neuron-specific alternative coding regions or, more commonly, longer 3′ untranslated regions (3′ UTRs) (reviewed in Mitschka and Mayr, 2022). The neural-enriched exons and ultra-long 3′ UTRs, herein referred to as “neuronal RNA signatures,” are a conserved feature of animal neurogenesis and have been found in animals from flies to humans (Barbosa-Morais et al., 2012; Brown et al., 2014; Hilgers et al., 2011; Miura et al., 2013; Smibert et al., 2012; Ulitsky et al., 2012). By increasing genetic versatility in cells that are particularly morphologically and functionally complex, neuronal RNA isoforms are thought to contribute to the robust coordination of neuronal processes (Hilgers, 2015; Miura et al., 2014; Pereira-Castro and Moreira, 2021; Turner et al., 2018). For example, dysregulation of neural splicing programs causes widespread neurological alterations in animal models and has been associated with human neurological conditions including autism spectrum disorder, intellectual disability, and neurodegenerative diseases (Furlanis and Scheiffele, 2018; Irimia et al., 2014; Nik and Bowman, 2019; Torres-Méndez et al., 2022).

The synthesis of neuronal RNA signatures depends on ELAV/Hu proteins (reviewed in Hilgers, 2022; Wei and Lai, 2022). The members of this highly conserved family of RNA-binding proteins (RBPs) constitute widely used markers for neuronal identity: in most animals studied to date, at least one ELAV/Hu protein is expressed in all post-mitotic neurons from inception and throughout development (Campos et al., 1985, 1987; Pascale et al., 2008; Robinow and White, 1988; Yao et al., 1993). ELAV/Hu proteins bind to U-rich regions in thousands of RNAs (Carrasco et al., 2020; Scheckel et al., 2016) and regulate diverse aspects of RNA metabolism, from co-transcriptional processing to mRNA stability and translation (Ince-Dunn et al., 2012; Mukherjee et al., 2011; Pascale et al., 2005; Tebaldi et al., 2018; Tiruchinapalli et al., 2008; Yokoi et al., 2017). At the co-transcriptional level, the role of ELAV/Hu proteins in the production of neuron-specific AS and APA has been demonstrated in flies (Carrasco et al., 2020; Hilgers et al., 2012; Koushika et al., 1996; Lisbin et al., 2001; Soller and White, 2003; Wei and Lai, 2022; Wei et al., 2020) and mammals (Grassi et al., 2018; Mansfield and Keene, 2012; Scheckel et al., 2016; Zhu et al., 2006). In *Drosophila*, the three ELAV/Hu paralogs appear consecutively in the course of development: found in neurons (FNE) expression follows ELAV’s during embryogenesis (Samson and Chalvet, 2003), whereas RBP9 is not detectable before larval stages (Kim and Baker, 1993). Notably, *Drosophila* ELAV regulates all sites of neuron-specific APA and thereby represents the central effector of the neuronal 3′ UTR landscape. ELAV also directly mediates neuron-specific splicing events (Carrasco et al., 2020; Lee et al., 2021).

ELAV/Hu proteins are crucial for neuronal differentiation, maturation, and maintenance of the nervous system (Akamatsu et al., 1999; Alizzi et al., 2020; Dell'Orco et al., 2020; Lim and Alkon, 2012; Tebaldi et al., 2018). In *Drosophila*, the loss of either of the two predominantly cytoplasmic ELAV/Hu proteins is viable but causes specific morphological and behavioral defects: *rbp9* mutant flies exhibit reduced locomotion and a shorter lifespan, while in *fne* mutants, mushroom body morphology, dendrite arborization, and male courtship performance are disrupted (Alizzi et al., 2020; Kim et al., 2010; Zaharieva et al., 2015; Zanini et al., 2012). In contrast, loss of the nuclear ELAV protein impairs development and results in embryonic lethality (Campos et al., 1985), highlighting the important role of neuronal transcript signatures in neurogenesis. Neuron-specific functions have been demonstrated for some individual ELAV-dependent signatures, especially in cases in which ELAV targets are affected at the protein-coding level. For example, the expression of the neuron-specific protein isoforms of the genes *erect wing* (*ewg*) and *neuroglian* (*nrg*) depends on ELAV-dependent APA (Koushika et al., 1996, 2000; Lisbin et al., 2001; Soller and White, 2003), and loss of ELAV-mediated AS of *Down syndrome cell adhesion molecule 1* (*Dscam1*) impairs axon outgrowth (Zhang et al., 2019). Neuronal 3′ UTRs have also been attributed specific functions: the ultra-long *prospero* 3′ UTR drives the upregulation of Prospero protein in larval neurons, which is critical for adult nervous system function (Samuels et al., 2020).

Despite the well-established importance of ELAV proteins in neuronal identity, and their known involvement in multiple neurological diseases (Mirisis and Carew, 2019; Yano et al., 2016), the connection between the molecular effects on the neuronal transcriptome and the drastic phenotypic consequences on neuronal and organismal physiology are not well understood. Studies have been complicated by the functional redundancy between ELAV/Hu proteins, which display a very high structural similarity and can interchangeably act on each other’s targets, even across species (Borgeson and Samson, 2005; Lee et al., 2020; Wei et al., 2020; Zaharieva et al., 2015). In flies, in which ELAV constitutes the sole predominantly nuclear member of the ELAV/Hu family, an elegant cellular strategy ensures the robustness of ELAV function and the integrity of neuronal signatures: exon-activated rescue (EXAR). In *elav* mutant embryos, the mRNA encoding the ELAV paralog FNE undergoes AS to include a mini-exon encoding a motif that promotes nuclear localization. Thus, activated nuclear FNE (nFNE) compensates for the loss of ELAV activity in that it restores neuronal signatures (Carrasco et al., 2020). However, the molecular rescue is only partial; phenotypically, nFNE expression is insufficient to prevent the lethality of *elav* mutant flies, none of which survive embryogenesis. This has led to the hypothesis that some crucial molecular signatures can only be mediated by ELAV *in vivo*, leaving the process of neurogenesis vulnerable to fluctuations of this essential effector.

Here, we dissect how ELAV proteins progressively shape the neuronal transcriptome and connect the expression of newly established mRNA isoforms with neuronal differentiation and nervous system integrity. We show that when ELAV is impaired, nFNE effectively rescues all neuronal RNA signatures and viability in late embryogenesis, demonstrating an important physiological role for EXAR. ELAV activity is indispensable at the very onset of neuronal differentiation, during which neuronal isoforms of a subset of axon guidance effectors are established. Loss of the early-onset neuronal signatures during this critical developmental window causes irremediable damage that cannot be rescued in later stages of neurogenesis.

## Results

### EXAR rescues neuronal signatures and lethality in ELAV-impaired embryos

**Figure 1:**
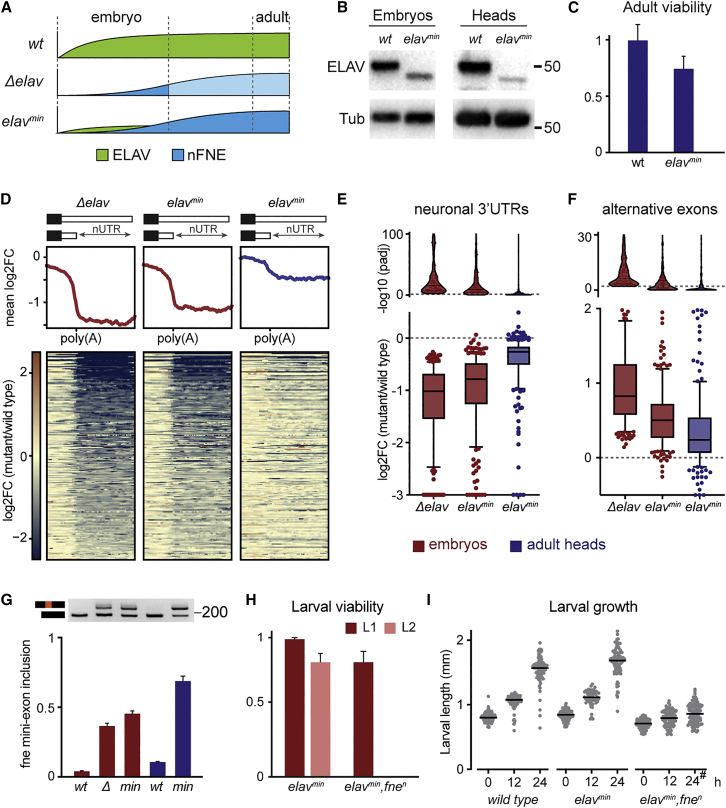
nFne rescues neuronal RNA signatures and embryonic lethality in an extreme *elav* hypomorph (A) Schematic representation showing, in each genotype, the progression of ELAV (green) and nFNE (blue) protein expression levels across the developmental trajectory. Light blue indicates predicted nFNE levels in the lethal *Δelav* genotype. (B) Expression of wild-type (WT) and truncated (*elav*^*min*^) ELAV protein in stage 16 (14–16 h AEL) embryos and adult heads. Tubulin served as a loading control. (C) Adult viability in flies of the indicated genotypes, measured as eclosion rate compared with expected Mendelian ratios. Eclosion rates were normalized to that of WT control flies. At least 500 flies were measured for each genotype. Error bars indicate mean ± SD of five biological replicates. (D) Heatmaps and profile plots centered around the proximal poly(A) site, showing deregulation and rescue of ELAV-dependent neuronal 3′ UTRs (nUTRs) in embryos (red) and fly heads (blue), compared with WT. (E and F) Global quantification (box plots) and corresponding adjusted p values (violin plots) of ELAV-dependent 3′ UTRs (E) and exons (F) in embryos (red) and adult heads (blue) of the indicated genotypes, compared with WT. Data points above the dotted lines in the violin plots represent significantly affected regions (adjusted p value [padj] < 0.01). (G) *fne* mini-exon inclusion in WT, *Δelav* (*Δ*), and *elav*^*min*^ (*min*), visualized by RT-PCR (top) and quantified by qRT-PCR (bottom). The expected RT-PCR product sizes are 232 (mini-exon included) and 187 bp (mini-exon excluded). Mini-exon inclusion was calculated as the ratio of *fne* mini-exon to total *fne* mRNA. Error bars indicate mean ± SD of three biological replicates. RNA was extracted from stage 16 (14–16 h AEL) embryos (red) or adult fly heads (blue). (H) Fraction of flies that successfully hatched into the first instar larval (L1) and L2 stages. 200 embryos (L1) and 100 L1 larvae (L2) were measured for each genotype. Error bars indicate mean ± SD of four biological replicates. (I) L1 growth measured as the head-to-tail length as a function of time (h after hatching). Hash indicates that all larvae were dead. Between 55 and 130 larvae were measured for each condition. See also Figure S1 and Table S1.

In the absence of ELAV, the activation of nFNE partially restores neuronal APA and AS through the EXAR mechanism (Carrasco et al., 2020); however, *elav* mutant flies only survive until the end of embryogenesis and fail to hatch into first instar larvae. This raises the question of whether EXAR is effective and physiologically relevant *in vivo*. We hypothesized that rather than rescuing the consequences of complete ELAV loss, EXAR may buffer variations of *elav* gene expression to promote nervous system development and function. To investigate this hypothesis, we designed a *Drosophila* model in which we aimed to activate EXAR without entirely abolishing ELAV function. Using CRISPR-Cas9 gene editing (Port and Bullock, 2016), we generated a frame-shift allele, *elav*^*min*^, encoding an ELAV protein in which the third RNA-recognition motif (RRM3) was disrupted (Figure S1A). As expected, we detected truncated ELAV protein in *elav*^*min*^ flies and also noted that it was expressed at very low—minute—levels throughout development and into adulthood (Figures 1A, 1B, and S1B).

Surprisingly, *elav*^*min*^ flies were viable and developed normally (Figure 1C), in stark contrast to *Δelav* null mutant flies that are embryonic lethal—suggesting that the essential ELAV functions are maintained despite near absence of the protein. To test this possibility, we quantified the outcome of ELAV molecular activity: production of neuron-specific 3′ UTRs and exons. We performed mRNA sequencing (mRNA-seq) in *elav*^*min*^ stage 16 (14–16 h after egg laying [AEL]) embryos and compared neuronal transcript signatures with those of *Δelav* null mutants. Interestingly, in *elav*^*min*^ embryos, ELAV-dependent 3′ UTRs were severely depleted and showed the expression drop downstream of the proximal poly(A) site characteristic of *Δelav* (Figures 1D and S1C; Table S1). Although *elav*^*min*^ embryos displayed a slim increase in the overall expression of neuronal signatures compared with *Δelav* (Figures 1E and 1F, bottom panels, and S1D–S1F), the number of significantly deregulated alternative 3′ UTRs and exons was comparable in *elav*^*min*^ and *Δelav* (Figures 1E and 1F, top panels), indicating limited functionality of the remaining truncated ELAV protein.

In *elav*^*min*^ adult flies, in contrast to embryos, most *fne* mRNAs included the mini-exon (Figure 1I). Moreover, ELAV-dependent 3′ UTRs and AS exons were expressed at near-normal levels in the brains of adult *elav*^*min*^ flies (Figures 1D–1F and S1F), indicating that EXAR functionality is fully deployed in the *elav*^*min*^ nervous system once development is completed. Therefore, we hypothesized that EXAR, through provision of nuclear ELAV activity by nFNE, rescues *elav*^*min*^ viability. We recombined *elav*^*min*^ with the previously characterized *fne*^*n*^ allele, in which removal of the *fne* mini-exon causes EXAR impairment through the expression of an obligatorily cytoplasmic FNE protein (Carrasco et al., 2020). Strikingly, *elav*^*min*^*,fne*^*n*^ mutants were 100% lethal, a phenotypic severity comparable to that of *Δelav* null mutants: all flies died within the first hours of the first-instar larval stage (Figures 1H and 1I). This result demonstrates that nFNE plays an important role in compensating for reduced ELAV function.

Taken together, our results show that in flies severely depleted of ELAV activity, EXAR restores neuronal signatures and viability even though it becomes activated later in development. We conclude that while residual ELAV activity in *elav*^*min*^ is sufficient to sustain essential functions until EXAR is deployed, supplementation by nFNE soon becomes critical for development.

### Induced nFNE can compensate for the complete absence of ELAV

**Figure 2:**
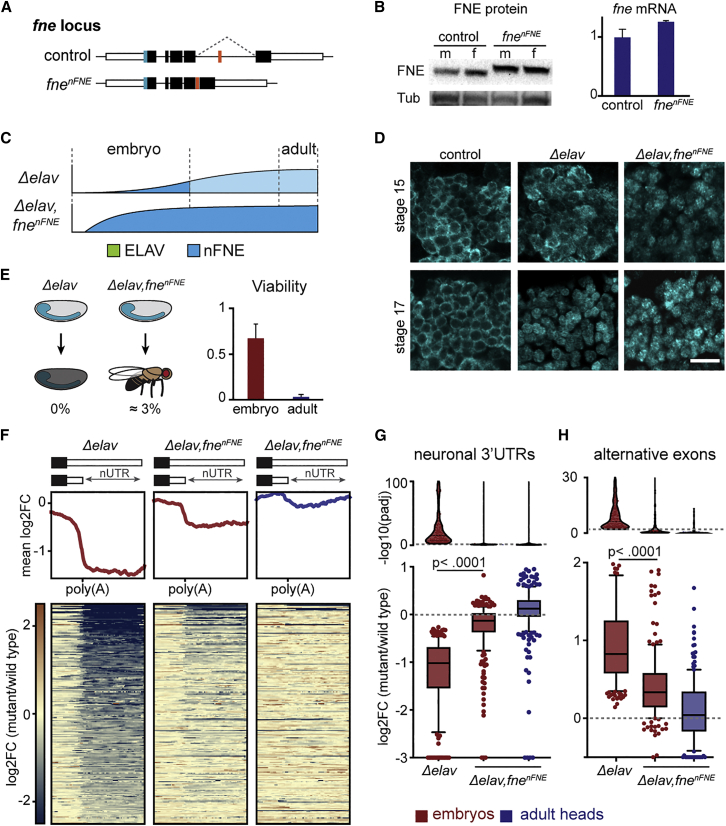
Precocious nFNE expression compensates for the complete absence of ELAV (A) Design of *fne*^*nFNE*^, a fly model expressing exclusively nuclear FNE. In control flies, the *fne* mini-exon is spliced out (dashed line). In *fne*^*nFNE*^, the deletion of flanking introns forces mini-exon inclusion. White, black, and orange boxes represent UTRs, constitutive coding exons, and the unannotated *n-fne* mini-exon, respectively. *fne* was endogenously FLAG tagged to allow for protein detection (blue box). (B) FNE protein and *fne* mRNA expression, in *fne*^*nFNE*^ and control (*fne*^*FLAG*^) fly heads. FNE expression, detected using a FLAG antibody, was comparable in males (m) and females (f). RNA levels were normalized to *RpL32* mRNA. Error bars indicate mean ± SD of four (embryos) or three (adult heads) biological replicates. (C) Schematic representation showing, in each genotype, the progression of ELAV (green) and nFNE (blue) protein expression levels across the developmental trajectory. Light blue indicates predicted nFNE levels in the lethal *Δelav* genotype. (D) Induced FNE nuclear localization in *fne*^*nFNE*^ neurons. Images are single confocal sections of the developing CNS in stage 15 (11–12 h AEL) and stage 17 (18–20 h AEL) embryos of the indicated genotypes. FNE was detected using an anti-FLAG antibody. Scale bar: 10 μm. (E) *fne*^*nFNE*^ partially rescues *elav* embryonic (red) and adult (blue) lethality. Viability was measured as the fraction of individuals that hatched into L1 (embryo) or eclosion rate compared with expected Mendelian ratios (adult). Eclosion rates were normalized to that of WT control flies. Error bars indicate mean ± SD of four (embryo) or five (adult) biological replicates. 200 embryos and 21 (*Δelav,fne*^*nFNE*^) or 778 (WT) adult flies were measured for each genotype. (F) Heatmaps and profile plots centered around the proximal poly(A) site show deregulation of ELAV-dependent nUTRs in *Δelav* embryos and rescue in *Δelav*,*fne*^*nFNE*^ embryos (red) and fly heads (blue), compared with WT. (G and H) Global quantification (box plots) and corresponding padjs (violin plots) of ELAV-dependent 3′ UTRs (F) and exons (G) in embryos (red) and adult heads (blue) compared with WT using RNA extracted from stage 16 (14–16 h AEL) embryos or adult fly heads. Data points above the dotted lines in the violin plots represent significantly affected regions (padj < 0.01). Statistical significance between genotypes was calculated using Friedman’s test for multiple comparisons. See also Figure S2 and Table S1.

Our finding that nFNE can perform most ELAV functions in adult flies raises the question of whether ELAV may be entirely expendable for embryonic viability if EXAR were activated sooner in the developmental trajectory. To test this possibility, we created a fly model of precocious EXAR: in the endogenous *fne* locus, we excised both introns flanking the mini-exon, thereby forcing exon inclusion and expression of the nFNE nuclear localization region (Figures 2A and S2A). The resulting *fne*^*nFNE*^ mutant fly expressed normal levels of *fne* mRNA and nFNE protein (Figure 2B) and displayed precocious FNE nuclear localization compared with endogenously regulated FNE in *Δelav* mutants (Figures 2C, 2D, and S2B). Strikingly, forced FNE activation rescued *Δelav* embryonic lethality by 67%; some *Δelav,fne*^*nFNE*^ flies even reached adulthood (Figure 2E).

Next, we tested nFNE’s capacity to perform ELAV’s molecular functions when activated early by analyzing ELAV target mRNAs in *Δelav,fne*^*nFNE*^ embryos and adult heads. We found that nearly all ELAV-dependent 3′ UTRs (Figures 2F, 2G, S2C, and S2F) and AS exons (Figures 2H, S2D, S2E, and S2G) were restored to wild-type levels in both embryos and adult flies, indicative of highly effective EXAR. Taken together, our results show that the progression of neurogenesis is directly dependent on ELAV function: higher levels of nuclear nFNE compensate for ELAV loss and result in significantly enhanced neuronal transcriptome signatures and developmental success.

### Impaired neuronal differentiation despite proper neuronal signatures

**Figure 3:**
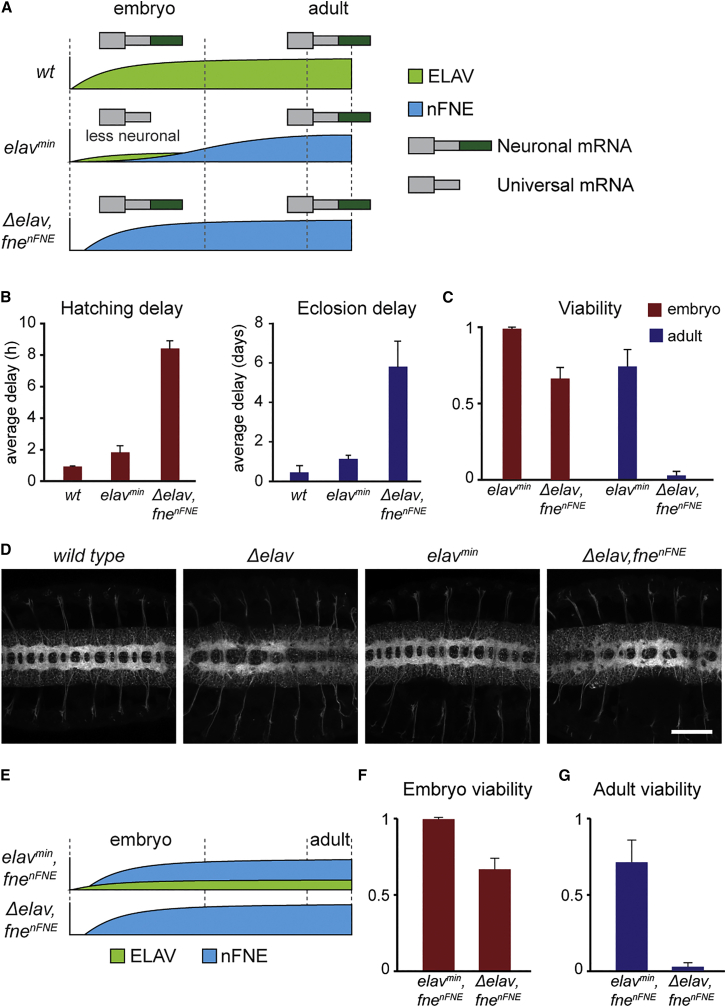
Impaired neuronal differentiation despite proper neuronal signatures (A) Schematic representation of the neuronal transcriptome and of the progression of ELAV (green) and nFNE (blue) protein expression levels across the developmental trajectory. Two models of EXAR are shown: endogenous (as in *elav*^*min*^ flies) and precocious (forced nuclear nFNE expression in *Δelav,fne*^*nFNE*^ flies). (B) Average delay in completing the indicated developmental transitions in each genotype compared with the median in the WT control. (C) Viability of each indicated genotype, measured as the fraction of individuals that hatched into L1 (embryo) or eclosion rate compared with expected Mendelian ratios (adult). Eclosion rates were normalized to those of WT control flies. Error bars indicate mean ± SD of four (embryos) and five (adult flies) biological replicates. 200 embryos and 515 (*elav*^*min*^), 21 (*Δelav,fne*^*nFNE*^), or 778 (WT) adult flies were measured for each genotype. Viability data for *elav*^*min*^ and *Δelav,fne*^*nFNE*^ are from Figures 1 and 2, respectively, and reproduced here for side-by-side comparison. (D) Axon scaffolds in the developing CNS of stage 16 (14 h AEL) embryos of the indicated genotypes, visualized by HRP immunohistochemistry. Shown are z stacks of multiple confocal sections. Scale bars: 50 μm. (E) Schematic representation of ELAV and nFNE levels across the developmental trajectory, in the flies shown in (F) and (G). (F and G) Viability in the indicated genotypes, measured as the fraction of individuals that hatched into L1 (F), and eclosion rate compared with expected Mendelian ratios (G). Eclosion rates were normalized to those of WT control flies. Error bars indicate mean ± SD of four (embryos) and five (adult flies) biological replicates. 200 embryos and 856 adult flies were measured for *elav*^*min*^*,fne*^*nFNE*^. Viability data for *Δelav,fne*^*nFNE*^ are from Figure 2 and reproduced here for side-by-side comparison with *elav*^*min*^*,fne*^*nFNE*^. See also Figure S3.

When we compared our EXAR fly models side by side, we noted a surprising disconnect between levels of neuronal signatures (represented schematically in Figure 3A) and physiological outcome: *Δelav,fne*^*nFNE*^ flies, despite carrying the full complement of neuronal signatures as embryos, were significantly developmentally delayed with reduced viability compared with *elav*^*min*^ (Figures 3B and 3C). Moreover, *Δelav,fne*^*nFNE*^ flies suffered very severe morphological phenotypes typical of impaired neuronal differentiation. The eyes of adult *Δelav,fne*^*nFNE*^ flies were dramatically reduced in size (Figure S3A); most notably, *Δelav,fne*^*nFNE*^ displayed the disorganization and reduced number of commissural axons characteristic for *Δelav* (Simionato et al., 2007; Zaharieva et al., 2015), while commissural axon guidance appeared normal in *elav*^*min*^ (Figures 3D and S3B).

We considered several possibilities to explain this apparent discrepancy between the molecular phenotype and the organismal-level physiological phenotype. By design, *Δelav,fne*^*nFNE*^ flies lack cytoplasmic FNE. Although endogenous FNE expression contributes to dendrite arborization in early larval stages (Alizzi et al., 2020), *fne* null mutants are homozygous viable and display much milder phenotypes than *Δelav,fne*^*nFNE*^ (Zanini et al., 2012). Thus, depletion of cytoplasmic FNE is unlikely to constitute a major determinant for the observed defects. Moreover, commissural axon patterning was normal in *elav*^*min*^*,fne*^*nFNE*^ embryos (Figure S3C), excluding a possible toxicity or other deleterious effect caused by the *fne*^*nFNE*^ allele or its protein product. Instead, we found that the ELAV^min^ protein critically contributes to neuronal differentiation: viability and development were restored in *elav*^*min*^,*fne*^*nFNE*^ flies compared with *Δelav,fne*^*nFNE*^ (Figures 3E and 3F). Therefore, residual truncated ELAV protein in *elav*^*min*^ executes a task that nFNE cannot: a cellular function essential for embryonic neuronal differentiation.

### ELAV activity is essential at the onset of neuronal differentiation

**Figure 4:**
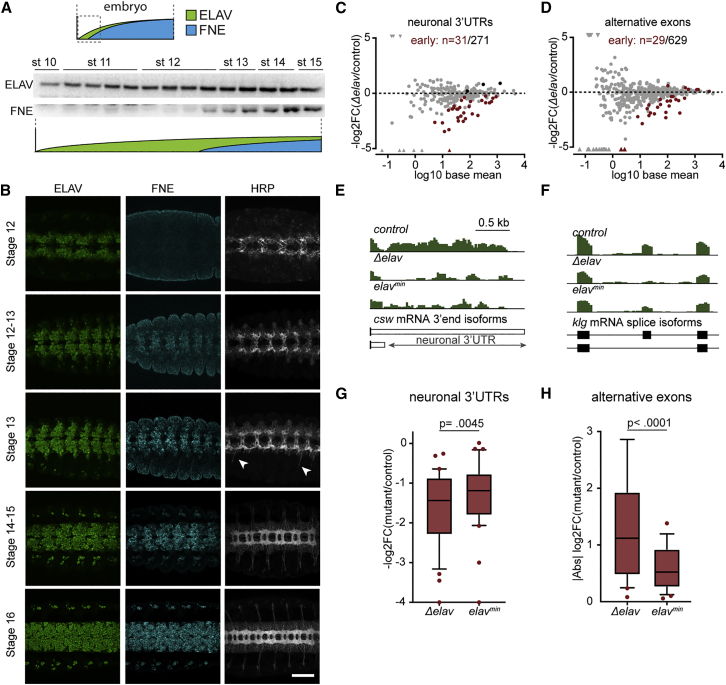
ELAV performs essential functions at the onset of neuronal differentiation (A and B) FNE expression is delayed compared with ELAV in the course of embryogenesis. (A) Time course western blot performed in 30-min intervals starting at 4:50 h AEL. 40 embryos (genotype *fne*^*nFNE*^) were used for each timepoint. ELAV (green) and nFNE (blue) protein expression levels across the developmental trajectory are represented schematically. (B) Single confocal sections (ELAV, FNE) or z stacks of multiple confocal sections (HRP) of the developing CNS in control embryos at the indicated developmental stages. White arrowheads indicate the emergence of motor neuron axons at stage 13. FNE was detected with an anti-FLAG antibody. Scale bars: 50 μm. The stage 16 HRP panel is reproduced from Figure 3D (WT). (C and D) Identification of differentiation-onset ELAV targets in stage 11–12 (7–8 h AEL) embryos. The MA plots represent the differential expression of ELAV-dependent 3′ UTRs (C) and alternatively spliced exons (D) in *Δelav* embryos compared with WT. Gray dots indicate all ELAV/FNE-dependent 3′ UTRs described in Carrasco et al. (2020) (C) and ELAV/FNE-regulated exons described in Carrasco et al. (2020) and Lee et al. (2021) (D). In red, targets significantly deregulated (padj < 0.01) in *Δelav* mutant embryos at 7–8 h AEL. (E and F) Representative examples (mRNA-seq tracks) of early ELAV-dependent targets, in which 3′ UTR (E) and exon (F) expression was rescued in *elav*^*min*^ compared with *Δelav* stage 11–12 embryos (7–8 h AEL). (G and H) Global quantification of early ELAV-dependent 3′ UTRs (E) and exons (F) in *elav*^*min*^ and *Δelav* compared with control (co; genotype: *fne*^*nFNE*^). To exclude confounding effects from the endogenous *fne* gene, *Δelav* and *elav*^*min*^ also carry the *fne*^*nFNE*^ allele, which is not expressed yet at 7–8 h AEL. Statistical significance between genotypes (stage 11–12 embryos, 7–8 h AEL) was calculated using Friedman’s test for multiple comparisons. See also Figure S4 and Table S2.

Our results above indicate that ELAV performs an essential function that cannot be compensated by nFNE—however, the inability of nFNE to fully rescue loss of ELAV is not intrinsic to the protein, since it seems to be able to act on most, if not all, targets of ELAV (Figure 1, adult neuronal signatures). Therefore, we reasoned that different developmental timing of ELAV activity versus nFNE expression may explain the incomplete rescue of *Δelav* mutants by nFNE. ELAV expression occurs as soon as post-mitotic neurons are born during the first wave of neurogenesis (Bier et al., 1988; Robinow and White, 1991) and precedes FNE expression in all neurons of the central and peripheral nervous system (Samson and Chalvet, 2003). In a time course experiment combining western blot and immunohistochemistry in developing *Drosophila* embryos, we detected ELAV in stage 10 embryos; expression rapidly increased to abundance by late stage 11. By contrast, FNE was undetectable until late stage 12 and was abundant in neurons only by stage 14 (Figures 4A, 4B, and S4A). Importantly, midline neuron axonogenesis begins during stage 13 (Jacobs and Goodman, 1989), while FNE expression is still low or absent (Figure 4B, top panels). This led us to hypothesize that lack of the earliest ELAV functions, at the onset of neuronal differentiation, causes permanent developmental defects and lethality.

To test this hypothesis, we characterized the molecular output of early-onset ELAV expression by comparing mRNA profiles of *Δelav,fne*^*nFNE*^ and *elav*^*min*^ embryos at stages 11–12 (7–8 h AEL), before FNE expression begins. We detected 31 neuronal 3′ UTRs (Figures 4C and 4E) and 29 alternative exons (Figures 4D and 4F) significantly affected in *Δelav* embryos, demonstrating that ELAV mediates neuronal APA and AS as soon as neurons are specified. In *elav*^*min*^ embryos, which only differ from *Δelav* in the expression of minute amounts of (truncated) ELAV, the expression of these early transcript signatures was significantly improved (Figures 4E–4H and S4B–S4E). Interestingly, restoration of early neural splicing patterns was more pronounced than that of APA patterns (Figures S5A–S5D). Considering the limitations in detecting neuron-specific RNAs in whole embryos at this early stage of development, we may be underestimating the actual number of early-onset ELAV-dependent RNA isoforms. In any case, the restoration of even very low levels of early ELAV expression in *elav*^*min*^ flies was sufficient to partially rescue molecular (neuronal APA and AS; Figures 4G and 4H) features, which then had an outsized impact on ameliorating the physiological (midline axonogenesis, developmental progression, and viability; Figures 3B–3D) phenotypes of ELAV loss.

Importantly, adult *elav*^*min*^ flies were short lived and displayed motor function defects (Figures S5E–S5G), suggesting that these flies suffered defects during development that manifested later. We infer that the improvement of differentiation-onset neuronal signatures in *elav*^*min*^ rescued gross morphological defects and embryonic viability but were insufficient to promote the formation of a fully functional nervous system. To challenge this hypothesis, we tested the possibility that adult *elav*^*min*^ phenotypes originated later than the early window of ELAV activity and dissected the contribution of early neuronal signatures from those rescued by nFNE in subsequent stages of embryogenesis. We supplemented *elav*^*min*^ flies with the *fne*^*nFNE*^ allele, which endows embryos with strong, precocious EXAR (Figure 2). Remarkably, *elav*^*min*^,*fne*^*nFNE*^ flies displayed defects of severity equal to those seen in *elav*^*min*^ (Figures S5E–S5G), showing that adult neurological defects were caused by lack of ELAV function at differentiation onset and not later.

### Full restitution of RNA signatures by nFNE restores neuron differentiation and function

**Figure 5:**
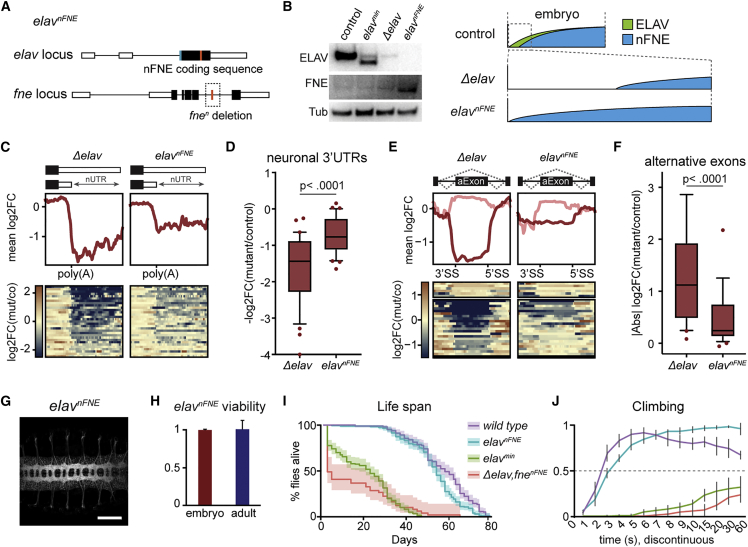
Early expression of nFNE rescues ELAV molecular and physiological function (A) Design of the *elav*^*nFNE*^ fly. The *nFNE* coding sequence was inserted into the *elav* locus, effectively replacing the *elav* coding sequence while preserving all *elav* regulatory elements; the endogenous *fne* gene lacks the mini-exon (*fne*^*n*^). White, black, and orange boxes represent UTRs, constitutive coding exons, and the *n-fne* mini-exon, respectively. *nFNE* was endogenously hemagglutinin (HA)-V5 tagged to allow for protein detection (blue box). (B) Western blot showing nFNE expression from the *elav* locus in *elav*^*nFNE*^ stage 11–12 (7–8 h AEL) embryos. FNE was detected with an anti-V5 antibody. 60 embryos were used for protein preparation for each genotype. ELAV (green) and nFNE (blue) protein expression levels across embryonic developmental time are represented schematically. The zoomed-in region represents the critical window of early-onset neuronal differentiation. Control genotype: *fne*^*nFNE*^. (C–F) Heatmaps and profile plots (C and E) and global quantification (D and F) of early-onset ELAV-dependent 3′ UTRs (C and D) and exons (E and F), showing deregulation in *Δelav* embryos and rescue of their expression in *elav*^*nFNE*^ embryos (stage 11–12, 7–8 h AEL). Light red and dark red lines represent up- and down-regulated 3′ UTRs and exons, respectively, compared with control (co; genotype: *fne*^*nFNE*^). To exclude confounding effects from the endogenous *fne* gene, *Δelav* and *elav*^*min*^ also carry the *fne*^*nFNE*^ allele, which is not expressed yet at 7–8 h AEL. Statistical significance between genotypes was calculated using Friedman’s test for multiple comparisons. (G) Axon scaffold in the CNS of a stage 15 (12 h AEL) embryo, visualized by HRP immunohistochemistry. Shown are z stacks of multiple confocal sections. Scale bar: 50 μm. (H) *elav*^*nFNE*^ flies displayed normal embryonic and adult viability. Viability was measured as the fraction of individuals that hatched into L1 (embryo) and eclosion rate compared with expected Mendelian ratios (adult). Eclosion rates were normalized to that of WT control flies. Error bars indicate mean ± SD of four (embryos) and five (adult flies) biological replicates. 200 embryos and 890 adult flies were measured. (I) Lifespan measurement in adult flies of the indicated genotypes. The percentage of live flies is represented as a function of time after eclosion (solid line) with associated confidence intervals (shaded area). Flies scored: n = 51 (*Δelav,fne*^*nFNE*^), 200 (*elav*^*min*^), 200 (*elav*^*nFNE*^), and 200 (WT). (J) Measurement of climbing performance in adult flies of each genotype shown as the fraction of flies found above the 5 cm mark at the indicated time points following startling. Error bars indicate mean ± SD of five biological replicates with 50 measurements each. Flies scored: n = 18 (*Δelav,fne*^*nFNE*^), 50 (*elav*^*min*^), 50 (*elav*^*nFNE*^), and 50 (WT). See also Figure S5 and Table S2.

To test if restoration of early-onset signatures could rescue all neurological phenotypes, we created a fly model in which EXAR occurred at differentiation onset. We generated the allele *elav*^*nFNE*^, in which we endogenously replaced the *elav* protein-coding region with that of *nFne*. To preserve cytoplasmic FNE expression, we replaced the endogenous *fne* allele with *fne*^*n*^, which encodes a nuclear translocation-deficient FNE (Figure 5A). Of note, these flies are entirely devoid of ELAV protein. Endowing *nFne* with *elav* transcriptional and post-transcriptional regulatory properties enabled early-onset nFNE expression, abundant by stages 11–12 (Figure 5B). This led to efficient expression of early-onset neuronal 3′ UTRs (Figures 5C and 5D) and alternative exons (Figures 5E and 5F); importantly, the molecular rescue was more successful than in *elav*^*min*^ (Figures S5A–S5D). In *elav*^*nFNE*^ embryos, axon patterns were fully restored (Figure 5G). Strikingly, not only were *elav*^*nFNE*^ flies 100% viable (Figure 5H), but we also discerned no morphological or behavioral phenotypes into adulthood. Lifespan and locomotor function of *elav*^*nFNE*^ flies were indistinguishable from wild type (Figures 5I and 5J). Taken together, our results demonstrate that the early establishment of the full complement of neuronal transcript signatures is crucial not only for the completion of embryogenesis but has far-reaching consequences long into adulthood. We also show that when expressed at the right time, nFNE can fully assume all physiological functions of ELAV. In situations where ELAV is depleted but not completely absent, EXAR plays an important physiological compensatory role—however, it cannot rescue complete loss of ELAV due to different developmental expression timelines.

Notably, the 60 early-onset ELAV-dependent RNA isoforms we identified among ELAV’s AS and APA targets are highly enriched in genes required for axon guidance, cell adhesion, and neurite pathfinding (Tables S1 and S2). Many include genes whose neuron-specific protein-coding isoform depends on ELAV (Figures S5A and S5B; Carrasco et al., 2020; Koushika et al., 1996; Koushika et al., 2000; Lisbin et al., 2001; Soller and White, 2003; Yu et al., 2009; Zhang et al., 2019) and whose collective loss likely causes the observed irremediable axonal defects. We conclude that establishment of neuronal transcriptome signatures specifically at the time of neuronal differentiation onset is a critical, limiting step in neuronal development that, if disrupted, cannot later be rescued by subsequent gain of ELAV function through EXAR.

## Discussion

The importance of ELAV/Hu proteins for neuronal differentiation and physiology has been known for decades. In contrast to the majority of other key regulators that top the hierarchy of developmental programs—typically transcription factors—nuclear ELAV/Hu proteins exert their function in neurogenesis by mediating the production of neural mRNA isoforms not seen in other cell types. The recent characterization of ELAV-dependent signatures genome wide (Carrasco et al., 2020; Lee et al., 2021) has brought to light the extent of ELAV’s influence on the coding and non-coding neuronal transcriptome and raised the question of how alternative exons and 3′ UTRs impact neurogenesis and neuron physiology (Wei and Lai, 2022).

We show that the ELAV-dependent deployment of neuronal signatures is required at the critical developmental window in which the first post-mitotic neurons are born. ELAV expression is most crucial at this stage, since the robustness mechanism that preserves neuronal signatures in the absence of ELAV, EXAR, is not active yet. The loss of what we term differentiation-onset neuronal signatures irremediably results in severe axonal defects and lethality (Figures 3 and 4). These signatures include coding exons of multiple genes required for axon outgrowth, including the cell adhesion genes *Nrg*, *Fasciclin 1*, and *Dscam1* (Table S2). Interestingly, deletion or suppression of the ELAV-dependent *Dscam1* isoform impairs axon projection (Yu et al., 2009; Zhang et al., 2019) but does not fully recapitulate the early loss of all neuronal signatures, indicating that *Dscam1* is an essential, but not exclusive, differentiation-onset target of ELAV. Some additional functional early targets likely went undetected. For example, we observed the neuronal 3′ UTR of the gene *commissureless* (*comm*) in late, but not early, embryos; however, the downregulation of Comm in *Δelav* mutants results in early differentiation defects (Simionato et al., 2007), indicating that *comm* may constitute a functional differentiation-onset APA target. Taken together, we propose that the cumulative deregulation of multiple early ELAV targets disrupts the initiation of the neuronal differentiation program.

Importantly, once the early, limiting step in neuronal differentiation is taken, ELAV-mediated AS and APA isoforms remain crucial for development and adult function. While low ELAV levels are sufficient to overcome differentiation onset in stages 11–12, soon high levels become necessary, as highlighted by the lethality of EXAR-deficient *elav*^*min*^*,fne*^*n*^ mutants (Figure 1). Of note, this and our previous work (Carrasco et al., 2020) identified a total of ∼400 protein-coding genes (Table S1) with an ELAV-dependent AS and/or APA isoform, most of which are “late.” For example, restitution of the ELAV-dependent protein variant of the transcription factor Ewg can rescue multiple, mostly late-differentiation, phenotypes of *elav* hypomorphic mutants such as synaptic growth (Haussmann et al., 2008), indicating that the *ewg* neuronal isoform contributes substantially to the phenotypic rescue seen in models of induced post-onset EXAR, such as *Δelav,fne*^*nFNE*^ (Figure 2). Our study demonstrates the relevance of EXAR as an endogenous mechanism that maintains the integrity and function of the nervous system in the case of insufficient ELAV activity. Our findings also shed some light on puzzling results from early studies: the partial viability of *elav*^*ts1*^ flies despite the near depletion of ELAV during neurogenesis (Campos et al., 1985; Samson et al., 1995) suggested that very little ELAV expression was sufficient to overcome embryonic lethality. We now demonstrate that in fact, high levels of ELAV function are essential for neuronal development and are provided by nFNE through EXAR when ELAV is impaired. Starting at about embryonic stage 15 in *Δelav* mutants (Carrasco et al., 2020), stage 13 in our model of precocious EXAR (*Δelav,fne*^*nFNE*^; Figure 2), and stages 11–12 in our model of differentiation-onset EXAR (*elav*^*nFNE*^; Figure 5), the activation of nFNE can fully replace ELAV function and rescue neuronal signatures for the remainder of the fly’s life. It is important to note that the third *Drosophila* ELAV/Hu paralog, RBP9, starts expressing in neurons during late larval or pupal stages (Kim and Baker, 1993). Therefore, while we show that FNE plays a major role in rescuing inadequate ELAV expression, it is possible that RBP9 is also involved in ensuring nuclear ELAV/Hu function and contributes to the rescue of post-embryonic *elav* phenotypes.

Interestingly, in *elav*^*min*^ flies, a truncated ELAV protein missing most of RRM3 was able to carry out crucial functions, such as the rescue of differentiation-onset molecular signatures. Previous reports showed that RRMs 1 and 2 are sufficient to bind RNA (Chung et al., 1996; Lisbin et al., 2000; Park et al., 2000; Wang et al., 2013). RRM3, while also mediating canonical RNA interactions (Pabis et al., 2019), plays a more important role in protein multimerization (Toba and White, 2008). In the *elav*^*min*^ background, in which ELAV levels are severely limiting, we observe that early-onset AS targets are more efficiently processed than APA targets (Figure 4). It is possible that RRM3 is required for efficient APA but not AS. Alternatively, the suppression of proximal poly(A) sites, but not splice sites, may require a higher number of RNA-binding ELAV molecules or depend on ELAV multimerization. A role for dimerization in APA has been demonstrated in structural studies of the tetrameric cleavage factor I(m) (CFI_m_) complex CFI_m_25/68, in which the concomitant binding of two CFI_m_25 subunits to two separate UGUA motifs facilitates RNA looping and inhibits poly(A) site usage (Yang et al., 2011). It is tempting to speculate that ELAV multimerization promotes proximal poly(A) site suppression through a similar mechanism.

ELAV/Hu proteins share very high sequence similarity, with largely identical RNA-binding regions. However, it was shown that *in vitro*, FNE binds to *ewg* RNA with less affinity than ELAV (Zaharieva et al., 2015), raising the possibility that subtle differences in binding affinity could impact the ability of FNE to compensate for ELAV. While it is possible that nFNE binds to ELAV targets with reduced affinity *in vivo* as well, we demonstrated in our *elav*^*nFNE*^ fly model that all molecular (transcript signatures) and physiological (phenotypic outcomes) functions of ELAV were fully rescued by nFNE when it was expressed in the correct developmental window (Figure 5). Our data assert that ELAV/Hu proteins can interchangeably act on the same molecular targets and that observed differences in *elav* molecular and physiological phenotypes are due to distinct spatiotemporal expression patterns and subcellular localization.

### Limitations of the study

In whole-embryo mRNA-seq, the signal corresponding to cell-specific transcript isoforms can be diluted by that of more broadly expressed isoforms, which limits the sensitivity of the detection and quantification of neuron-specific sequences. This is particularly relevant for lowly expressed neuronal 3′ UTRs and for AS isoforms of widely expressed genes. Therefore, the actual number of ELAV targets, in particular of early-onset targets, is likely higher than that indicated in this study. In experiments that measured the viability of *Δelav,fne*^*nFNE*^ flies, the number of assessed animals was limited. Obtaining viable progeny from *Δelav,fne*^*nFNE*^ males proved difficult due to their low viability, short lifespan, and severe motor defects.

## STAR★Methods

### Key resources table

**Table:** 

REAGENT or RESOURCE	SOURCE	IDENTIFIER
**Antibodies**		

Rabbit polyclonal anti-ELAV	Carrasco et al. (2020)	N/A
Mouse anti-FLAG	Sigma-Aldrich	Cat#F1804; RRID:AB_262044
Mouse anti-V5	Thermo Fisher Scientific	Cat#MA5-15253; RRID:AB_10977225
Mouse anti-Tubulin	Developmental Studies Hybridoma Bank	Cat#4A1; RRID:AB_2732839
HRP-conjugated mouse anti-FLAG	Sigma-Aldrich	Cat#A8592; RRID:AB_439702
HRP-conjugated anti-rabbit	Cell Signaling Technology	Cat#7074; RRID:AB_2099233
HRP-conjugated anti-mouse	Sigma-Aldrich	Cat#A9044; RRID:AB_258431
Alexa Fluor® 488 goat anti-rabbit IgG	Invitrogen	Cat#A11008; RRID:AB_143165
Alexa Fluor® 555 goat anti-mouse IgG	Invitrogen	Cat#A28180; RRID:AB_2536164
Alexa Fluor® 647 goat anti-HRP	Jackson ImmunoResearch Labs	Cat#123-605-021; RRID:AB_2338967

**Critical commercial assays**		

Stranded mRNA Prep kit	Illumina	Cat#20040534

**Deposited data**		

Raw and analyzed sequencing data	This paper	GEO: (GSE199714)
Raw imaging data	This paper	Mendeley data: https://doi.org/10.17632/d96s2fxm5t.1

**Experimental models: Organisms/strains**		

*D. melanogaster:* wild type: w^118^	Bloomington Drosophila Stock Center	BDSC:5905; RRID:BDSC_5905
*D. melanogaster:* GFP-marked FM7 balancer: FM7i, P{w[+mC] = ActGFP} JMR3/C(1)DX, y[1] f[1]	Bloomington Drosophila Stock Center	BDSC:4559; RRID:BDSC_4559
*D. melanogaster:* GFP-marked FM7 balancer: y^1^ w^∗^ N^1^/FM7 c, P{w^+mC^ = GAL4-twi.G}108.4, P{UAS-2xEGFP}AX	Bloomington Drosophila Stock Center	BDSC:6873; RRID:BDSC_6873
*D. melanogaster: elav* deletion: elav^CDS20^	Carrasco et al. (2020)	N/A
*D. melanogaster: fne*^*n*^	Carrasco et al. (2020)	N/A
*D. melanogaster: fne*^*FLAG*^	Carrasco et al. (2020)	N/A
*D. melanogaster: elav*^*min*^	This paper	N/A
*D. melanogaster: fne*^*nFNE*^	This paper	N/A
*D. melanogaster: elav*^*nFNE*^	This paper	N/A

**Oligonucleotides**		

Oligonucleotides used for qRT-PCR	Table S3	N/A
Primer fne/nFne Forward: CGCCAACAA TCCGAGCAATA	Carrasco et al. (2020)	N/A
Primer fne/nFne Reverse: AGTCATGGCA TTTCCCGGTA	Carrasco et al. (2020)	N/A

**Recombinant DNA**		

Homology donor plasmid to generate *fne*^*nFNE*^ CRISPR flies	Table S3	pJet1.2-nFNE
Homology donor plasmid to generate *elav*^*nFNE*^ CRISPR flies	Table S3	pJet1.2-elav-nFNE
Guide RNA expression plasmid used to generate *elav*^*nFNE*^ CRISPR flies	Carrasco et al. (2020)	pCFD5-elav^CDS^gRNAs
Guide RNA expression plasmids	Port and Bullock (2016)	pCFD5; RRID:Addgene_73914

**Software and algorithms**		

R 4.1.1	R Core Team (2021)	https://CRAN.R-project.org
dplyr_1.0.7	Wickham et al. (2022)	https://dplyr.tidyverse.org
rtracklayer_1.44.2	Lawrence et al. (2009)	https://github.com/lawremi/rtracklayer
GenomicFeatures_1.36.4	Lawrence et al. (2013)	https://github.com/Bioconductor/GenomicFeatures
DEXSeq_1.38.0	Anders et al. (2012)	http://bioconductor.org/packages/release/bioc/html/DEXSeq.html
deeptools 3.5.0	Ramírez et al. (2016)	https://github.com/deeptools/deepTools
bedtools2	Quinlan and Hall (2010)	https://github.com/arq5x/bedtools2
snakePipes 2.5.1	Bhardwaj et al. (2019)	https://github.com/maxplanck-ie/snakepipes
featureCounts 2.0.0	Liao et al. (2019)	https://subread.sourceforge.net
HTSeq	Anders et al. (2015)	https://github.com/simon-anders/htseq
exaR	Carrasco et al. (2020)	https://github.com/hilgers-lab/apa_target_caller
survival 3.3-1	Therneau (2022)	https://CRAN.R-project.org/package=survival
GraphPad Prism (version 8)	N/A	https://www.graphpad.com

### Resource availability

#### Lead contact

Further information and requests for resources and reagents should be directed to and will be fulfilled by the lead contact, Valérie Hilgers (hilgers@ie-freiburg.mpg.de).

#### Materials availability

The newly generated Drosophila strains are available from the lead contact on request.

### Experimental model and subject details

#### Drosophila melanogaster

Experiments in this study used male *Drosophila melanogaster*. For technical reasons, mutant males -but not females- could be obtained in sufficient amounts to perform experiments, thus the influence of sex could not be investigated. Experiments were performed in different stages of embryonic, larval and adult development; the exact stage is reported in each figure legend. Flies were raised and maintained at 25°C following standard fly husbandry protocols. *w*^*1118*^ control flies and GFP-marked balancer chromosomes were obtained from the Bloomington stock center (lines 5905, 4559, and 6873, respectively). Flies denoted as *Δfne* are of the genotype *Df(1)fne* and were obtained from Matthias Soller (Zaharieva et al., 2015). CRISPR-Cas9 genome editing followed the procedure described in (Port and Bullock, 2016). Embryo injections were performed by Bestgene, Inc. To generate the *elav*^*min*^ mutant, one guide RNA (GCTGCCCTGTGGCAGCTGTT) targeted the *elav* coding region and non-homologous end joining repair introduced an indel at Q420, resulting in a shift in the ELAV ORF (ORF +2). To generate the *fne*^*nFNE*^ fly, two guide RNAs (TAATACGAACCTAATGCGAC, GCACTTTAGGTACTCACCGC) and a template sequence were designed to replace the endogenous intron containing the n-fne mini-exon, by the n-fne mini-exon without the adjacent intronic sequence; the two guide RNAs, the 1.09-kb donor homology sequence and DNA encoding Cas9 were co-injected into *fne*^*FLAG*^ (Carrasco et al., 2020) embryos to produce an N-terminally FLAG-V5 tagged protein. To generate *elav*^*nFNE*^, in which the *elav* coding region was replaced by the nFNE coding region, two guide RNAs previously used to generate an *elav* protein null mutant (TCCATTTGGGCCGCTCTACT, GTCTACTCCGCCGCCAGCTC, (Carrasco et al., 2020)) were co-injected with 2.57 kb of template sequence containing the upstream *elav* intronic region, the first 12 nt of the *elav* coding exon in frame with an HA-V5 tag sequence, the nFNE cDNA and the proximal region of *elav* 3′ UTR. The same guide RNAs and donor sequence were also injected into *fne*^*n*^ embryos (Carrasco et al., 2020) to generate *elav*^*nFne*^*, fne*^*n*^. Sequences used as template for genome editing can be found in Table S3.

### Method details

#### Embryo collection

All flies used in this study were raised in heterozygosis with GFP-marked balancer chromosomes. Embryos were collected on agar plates and aged for the appropriate time at 25°C. Embryos were dechorionated following standard procedures and placed on a plate containing halocarbon oil. Male mutant embryos were selected according to embryonic age, morphology, and against GFP signal.

#### Quantification of developmental transitions

Embryonic viability was calculated as the fraction of embryos that successfully completed embryogenesis and hatched into first instar larvae. Dechorionated embryos coated with halocarbon oil were placed on agar plates. Starting at 20–22 h AEL, the count of unhatched embryos was recorded every two hours for fourteen hours and one last time at 46–48 h AEL. Hatching delay was calculated as the difference in hatching time between a given embryo and that of the median time in the control (wild type) group.

Recently-hatched L1 larvae were transferred to agar plates containing yeast paste (2:3 ratio reconstitution of dry yeast in water). Larvae were kept at 25°C for 48 h, after which the survival and developmental stage of each larva was assessed visually using a stereoscope. To measure larval growth, recently-hatched L1 larvae were placed in agar plates coated with a fine layer of yeast solution (2:4 ratio reconstitution of dry yeast in water) and allowed to age in a humid chamber at 25°C. Images of crawling or dead larvae were captured using a Leica M165FC stereoscope and head-to-tail length was measured using *ImageJ*.

Adult viability was calculated as the fraction of mutant flies from a heterozygous stock compared to expected Mendelian ratios and normalized to ratios in the control (wild type) group. Virgin females heterozygous for a given mutation were crossed with males hemizygous for a balancer chromosome and were allowed to lay eggs for 24 h. Afterward, flies were discarded and eggs were allowed to develop for ten days at 25°C. The genotypes of eclosed flies - including those dead shortly after eclosion - were recorded every day for ten days. Hence, for genotypes with very low viability, like *Δelav,fne*^*nFNE*^, the number of scored adult flies appears very low. Eclosion delay was calculated as the difference in eclosion time between a given fly and that of the median time in the control group.

#### Adult life span measurement

For each genotype, 200 males were collected 48 h after eclosion and equally distributed into four large vials with food. For *Δelav,fne*^*nFNE*^ flies, due to very low viability that precludes harvesting high numbers of age-matched flies, 49 males were collected and distributed in three vials. Flies were transferred to fresh food and the number of dead flies was recorded every 48 h until all flies had died. Survival probability was calculated using the *survival* package in R and plotted using the *ggsurvplot* function from *survminer* (Therneau, 2022).

#### Negative geotaxis assay

Flies 3–5 days after eclosion were transferred to empty vials at a density of ten flies per vial. Flies were tapped down to the bottom of the vial for 3 s and allowed to freely move during 2 min. Movement was video recorded and the position of each fly with respect to the 5 cm line was determined at sequential timepoints. Each biological replicate was tapped 5 times separated by 2 min of climbing and recovery time.

#### Western blot

Protein detection was carried out using rabbit anti-ELAV (Carrasco et al., 2020), mouse anti-Tubulin (DSHB 4A1), mouse anti-V5 (Thermo Fisher Scientific MA5-15253) and peroxidase-conjugated mouse anti-FLAG (Sigma A8592) at concentrations 1:1000, 1:100, 1:8000 and 1:10000, respectively. Secondary peroxidase-conjugated antibodies (anti-rabbit (Cell Signaling Technology, #7074) and anti-mouse (Sigma A9044)) were used at 1:5000. For each sample, 12 heads or 40 embryos were homogenized in 50 μL PBS, subsequently mixed with 4xLDS sample buffer (Invitrogen NP0007) 0.2 M DTT and boiled 5 min at 95°C.

#### Immunohistochemistry

Staged sorted or unsorted embryos were fixed following standard protocols. Briefly, dechorionated embryos were fixed in 1:1 solution of heptane and 1× PBS, 50 mM EGTA, 4% PFA for 20 min. They were subsequently devitallinized, extensively washed from residual fixative and rehydrated. Prior to immunostaining, embryos were incubated for an hour in blocking solution (PBS 0.2% triton X-100, 5% BSA). Primary antibodies were used at concentration of 1:200 (rabbit anti-ELAV and mouse anti-FLAG (Sigma F1804)) and incubated together with fluorophore-conjugated anti-HRP 1:100 (Jackson ImmunoResearch Laboratories, Inc 123-605-021) overnight at 4°C. Other fluorophore conjugated secondary antibodies were used at concentrations 1:500 and incubated for 1 h at room temperature. Confocal imaging was performed on a Zeiss LSM 780 microscope.

#### RNA extraction and RT-PCR

Total RNA was extracted from biological samples using 12 heads or 15 embryos per condition in biological triplicates and quadruplicates, respectively. Samples were homogenized directly in TRIzol (Invitrogen) and RNA was extracted following the manufacturer’s instructions. RNA integrity was assessed using the 2100 Bioanalyzer (Agilent Technologies). 300 ng of total RNA was used for reverse transcription using iScript gDNA Clear cDNA Synthesis Kit (Bio-Rad). qRT-PCR was performed in a LightCycler 480 II instrument using FastStart SYBR Green Master (Roche). qRT-PCR primer sequences are listed in Table S3. For RT-PCR, cDNA was used as template for PCR amplification using GoTaq Hot Start Polymerase (Promega) following the manufacturer’s instructions. Primers against exons 5 and 6 (CGCCAACAATCCGAGCAATA, AGTCATGGCATTTCCCGGTA) of *fne-RA* were used to detect *fne* alternative splicing. DNA fragments were resolved in a 2% agarose gel.

#### mRNA sequencing and data processing

100 ng of total RNA were used to prepare mRNA-seq libraries using the Stranded mRNA Prep reagents (Illumina, 20040534) according to the manufacturer’s instructions. Paired-end sequencing was performed using the NovaSeq6000 (Illumina) and 150-bp or 100-bp reads for data shown in Figures 1 and 2, and Figures 4 and 5, respectively. Sequencing data were processed using the RNA-seq module from snakePipes (Bhardwaj et al., 2019) with default parameters. Quality control of mRNA-seq reads was done using FASTQC (Andrews, 2010).

#### 3′ UTR quantification

Differential expression of neuronal 3′ UTRs was carried out as in (Carrasco et al., 2020). Briefly, *featurecount* (Liao et al., 2019) was used to count the number of reads overlapping each of the previously re-annotated neuronal-isoform-aware 3′ UTR nodes and differential expression changes were subsequently quantified using DEXSeq (Anders et al., 2012). Nodes extracted from unique neuronal 3′ UTRs were merged and their fold change averaged following length normalization. Significance cut-offs of padj <0.01 and negative fold change were used to define neuronal 3′ UTRs affected in *elav* mutants in the different developmental timepoints. Seven previously identified ELAV 3′ UTR targets - *FBgn0037698*, *FBgn0003175*, *FBgn0001122*, *FBgn0264001*, *FBgn0261822*, *FBgn0053100*, *FBgn0031100* - were found to be false-positives due to variable genetic background in different fly strains and were thus excluded from this analysis.

#### Quantification of alternative exon usage

Reads mapping exons in the reference transcriptome (dm6, Ensembl release 96) were counted using HTSeq (Anders et al., 2015). Potential ELAV-dependent exons were obtained by combining exon regions from (Carrasco et al., 2020) and (Lee et al., 2021) and subsetting unique regions overlapping annotated exon nodes. In total, 629 exon regions were considered. Significance cut-offs of padj <0.01 were used to define ELAV-dependent 3′ UTRs affected in *elav* mutants in the different developmental timepoints.

#### Heatmaps

Fold change expression in ELAV-dependent neuronal 3′ UTRs and alternatively spliced exons were visualized using *plotHeatmap* from deepTools (Ramírez et al., 2016). Exons smaller than the visual resolution limit (bin size = 10 nt) were excluded from heatmaps.

### Quantification and statistical analysis

Statistical parameters and tests, sample sizes and number of biological and/or technical replicates are reported in the respective figure legends. Boxplots display boxes with interquartile ranges from first to third quartile; whiskers represent the 10^th^ and 90^th^ percentiles. Statistical tests and data visualization were performed, unless otherwise indicated in the respective methods section, using GraphPad Prism 8 for macOS.

## Data Availability

• mRNA sequencing data generated during this study have been deposited at NCBI Gene Expression Omnibus and are publicly available as of the date of publication. Accession numbers are listed in the Key resources table. Original western blot images and raw microscopy data have been deposited in Mendeley Data and are publicly available as of the date of publication. The DOI is listed in the Key resources table. • This paper does not report original code. • Any additional information required to reanalyze the data reported in this paper is available from the lead contact upon request.

## Supplemental information

Document S1. Figures S1–S5 and Table S2

Table S1. ELAV APA and AS targets, related to Figures 1, 2, S1, and S2

Table S3. qRT-PCR oligonucleotides and CRISPR homology sequences used in this study, related to Figures 1 and S1 and STAR Methods

Document S2. Article plus supplemental information
