# Supplementary material for: A critical developmental window for ELAV/Hu-dependent mRNA signatures at the onset of neuronal differentiation
Source: Cell Rep. 2022 Oct 25;41(4):111542. doi: 10.1016/j.celrep.2022.111542 (PMC9631114; doi:10.1016/j.celrep.2022.111542)
Supplement: Document S1. Figures S1–S5 and Table S2 [file mmc1.pdf]

Cell Reports, Volume *41*

Supplemental information

A critical developmental window for ELAV/Hu-dependent mRNA signatures at the onset of neuronal differentiation

Judit Carrasco, Fernando Mateos, and Valérie Hilgers

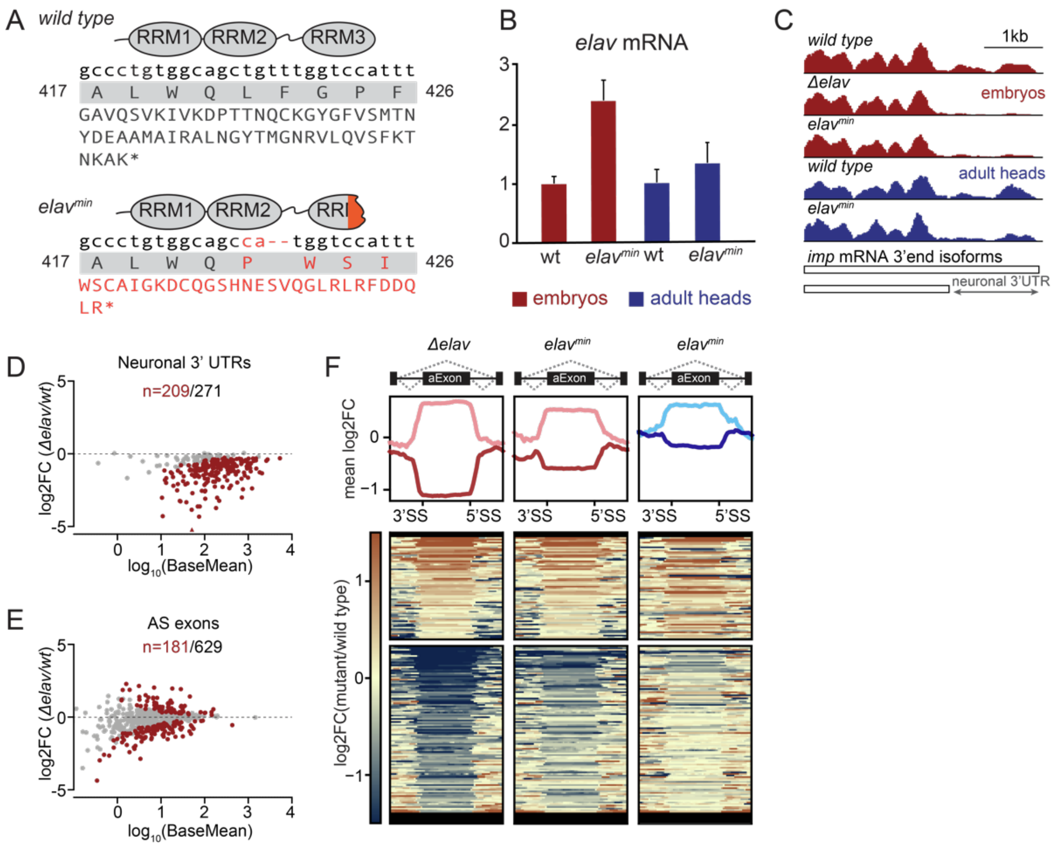

Figure S1. nFNE rescues neuronal RNA signatures in an extreme *elav* hypomorph. Related to Figure 1.

(A) Design of the new hypomorphic allele *elav^{min}*. Schematic representation of the ELAV protein, nucleotide sequence, and amino acid sequence in wild-type and *elav^{min}* flies. Sequences differing from wild type are in red. Four nucleotides are mutated in *elav^{min}*: a two-nucleotide substitution (TG to CA) followed by a two-nucleotide deletion (TT). The deletion in the *elav* open reading frame causes a frame-shift and premature stop codon, resulting in a truncation of the third RNA recognition motif (RRM3) of ELAV.

(B) *elav* is not downregulated at the mRNA level in *elav^{min}* compared to wild-type flies. Shown is the qRT-PCR quantification of *elav* mRNA expression in the indicated genotypes and developmental stages. Error bars indicate mean ±SD of four and three biological replicates of stage 16 (14-16h AEL) embryos (red) or adult fly heads (blue), respectively.

(C) Representative example (mRNA-seq tracks) of an ELAV target gene, *imp*, in which neuronal 3' UTR expression was rescued by EXAR in *elav^{min}* adult flies, but not in embryos.

(D, E) Identification of 209 ELAV-dependent 3' UTRs (D) and 181 alternatively spliced exons (E) in stage 16 (14-16h AEL) embryos. The MA plots represent the differential expression in *Δelav* embryos compared to wild type (wt). Grey dots indicate all ELAV/FNE-dependent 3' UTRs described in Carrasco et al., 2020 (D) and ELAV/FNE-regulated exons described in Carrasco et al., 2020 and Lee et al., 2021 (E). In red, targets

significantly deregulated (padj < 0.01) in *Δelav* mutant embryos at 14-16h AEL. These targets are used in Fig. 1 and Fig. 2 as ELAV-dependent targets.

(F) Rescue of ELAV activity in *elav*^{min} flies. Heatmaps and profile plots of ELAV-dependent exons in *elav*^{min} embryos (red) and fly heads (blue). Light and dark color lines represent up- and down-regulated exons, respectively. RNA was extracted from adult 3-day-old flies and stage 16 (14-16h AEL) embryos.

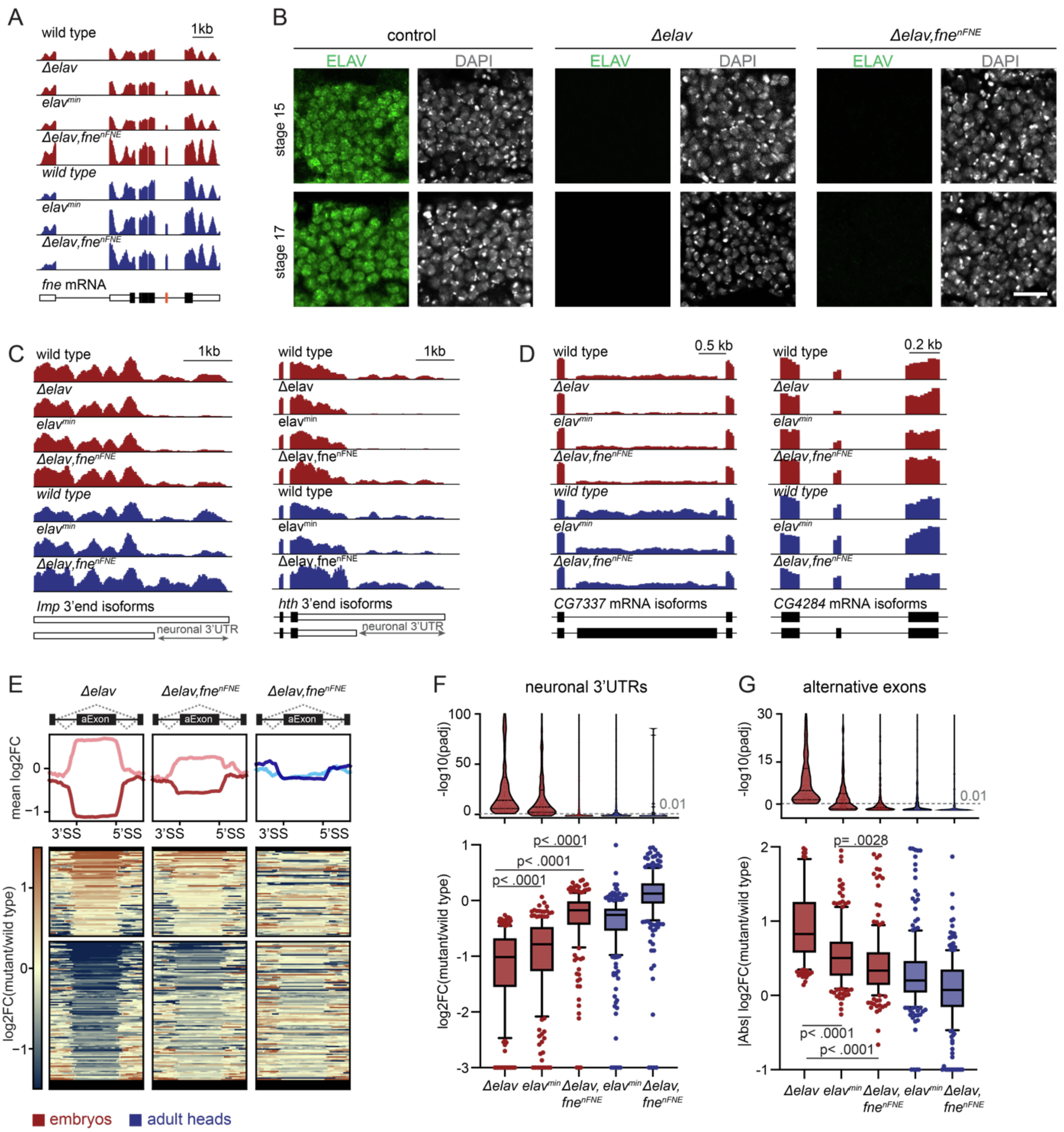

Figure S2. Induced EXAR can rescue ELAV molecular function. Related to Figure 2.

(A) mRNA-seq tracks showing *n-fne* mini-exon inclusion in stage 16 (14-16h AEL) embryos (red) and adult heads (blue) of the indicated genotypes. The orange box in the gene model represents the *n-fne* mini-exon.

(B) Additional imaging channels, corresponding to ELAV immunostaining and DAPI staining, of the confocal sections shown in Fig. 2D. Images are of the developing CNS in stage 15 (11-12h AEL) and stage 17 (18-20h AEL) embryos of the indicated genotypes. Scale bar: 10 μm.

(C, D) Representative examples of genes showing ELAV-dependent 3' UTR expression (C) and alternative exon inclusion (D) in stage 16 (14-16h AEL) embryos (red) and adult heads (blue) of the indicated genotypes.

E) Heatmaps and profile plots showing de-regulation of ELAV-dependent exons in *Δelav* embryos, and rescue in *fne*^{nFNE} embryos (red) and adult heads (blue). Light and dark color lines represent up- and down-regulated exons, respectively.

(F, G) Global quantification (box plots) and corresponding adjusted *p*-values (violin plots) of ELAV-dependent 3' UTRs (F) and exons (G) in embryos (red) and adult heads (blue) of the indicated genotypes compared to the wild-type control (wt). Statistical significance between genotypes was calculated using Friedman's test for multiple comparisons. Data are from Fig. 1F-G and Fig. 2G-H and shown side-by-side to allow for direct comparison. RNA was extracted from adult 3-day-old flies and stage 16 (14-16h AEL) embryos.

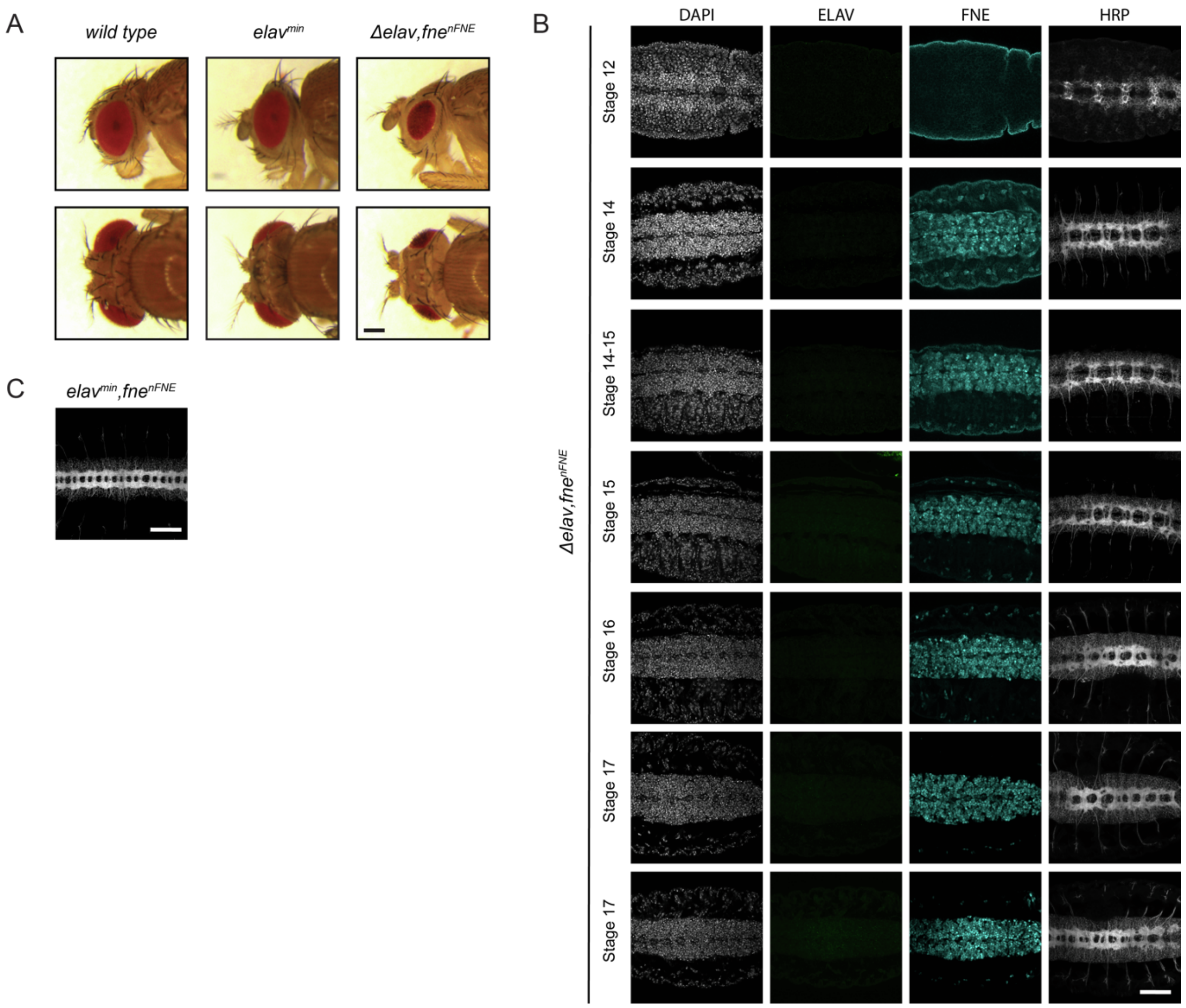

Figure S3. Impaired neuronal differentiation despite proper neuronal signatures. Related to Figure 3.

(A) Representative images of adult flies of the indicated genotypes. *Δelav,fne*^{nFNE} flies display a fully penetrant rough-eye phenotype, while wild-type control and *elav*^{min} flies do not. Scale bar: 200 µm.

(B) Axonal phenotype in the developing nervous system of *Δelav,fne*^{nFNE} flies. Shown are single confocal sections (DAPI, ELAV, FNE) or Z-stacks of multiple confocal sections (HRP) of the developing CNS at the indicated embryonic stages. FNE was detected with an anti-FLAG antibody. Scale bar: 50 µm. The stage 16 HRP panel is reproduced from Fig. 3D (*Δelav,fne*^{nFNE}).

(C) Axon scaffold in the developing CNS of a representative *elav*^{min}*,fne*^{nFNE} embryo at stage 16 (14-16h AEL), visualized by HRP immunochemistry. Shown is a Z-stack of multiple confocal sections. Scale bar: 50 µm.

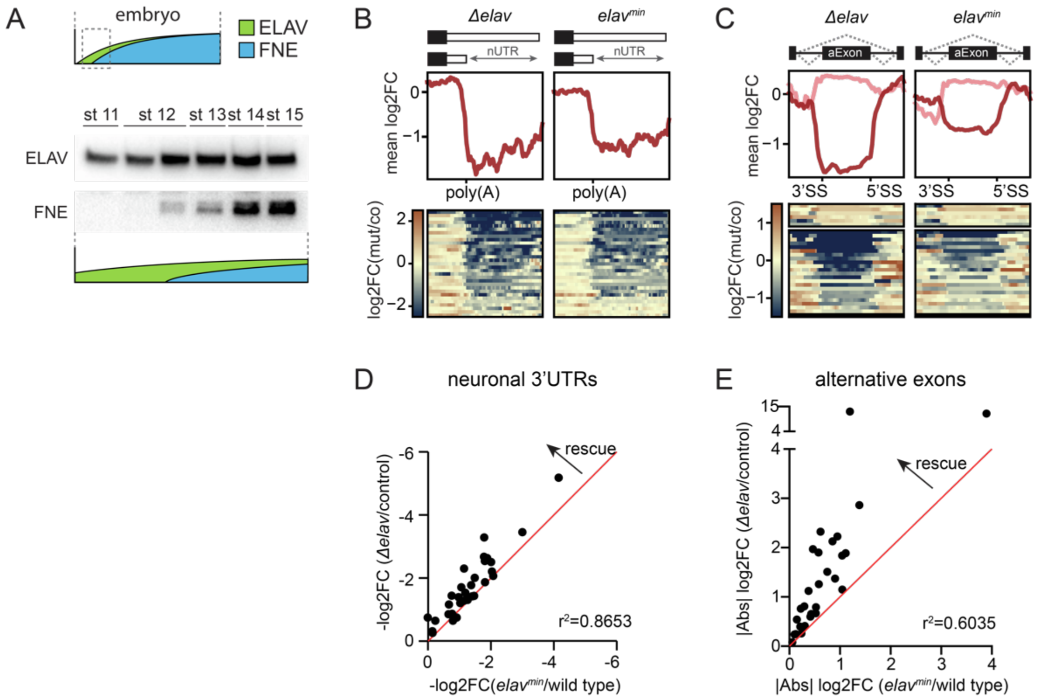

Figure S4. Rescue of early ELAV molecular signatures in *elav^{min}*. Related to Figure 4.

(A) Time-course Western Blot performed in 60-minute intervals starting at 6:20 h AEL. 40 embryos (genotype *fne^{FLAG}*) were used for each timepoint. ELAV (green) and nFNE (blue) protein expression levels across the developmental trajectory are represented schematically. FNE was detected with an anti-FLAG antibody.

(B, C) Heatmaps and profile plots of early-onset ELAV-dependent 3' UTRs (B) and alternative exons (C) showing deregulation in *Δelav*, and rescue of their expression in *elav^{min}* embryos compared to the wild-type control (stage 11-12, 7-8h AEL). Light red and dark red lines represent up- and down-regulated 3' UTRs and exons, respectively, compared to control (co; genotype: *fne^{nFNE}*). To exclude confounding effects from the endogenous *fne* gene, the genotypes *Δelav* and *elav^{min}* also carry the *fne^{nFNE}* allele, which is not expressed yet at 7-8h AEL.

(D, E) Scatter plots showing the differential expression of ELAV-dependent 3' UTRs (D) and alternative exons (E) in elav^{min} (x axis) and *Δelav* (y axis) stage 11-12 (7-8 h AEL) embryos compared to the wild-type control (wt). The shift towards the y axis indicates rescue of neuronal signatures in *elav^{min}*. For visualization purposes, fold-changes in down-regulated exons were transformed to absolute values and represented together with up-regulated exons.

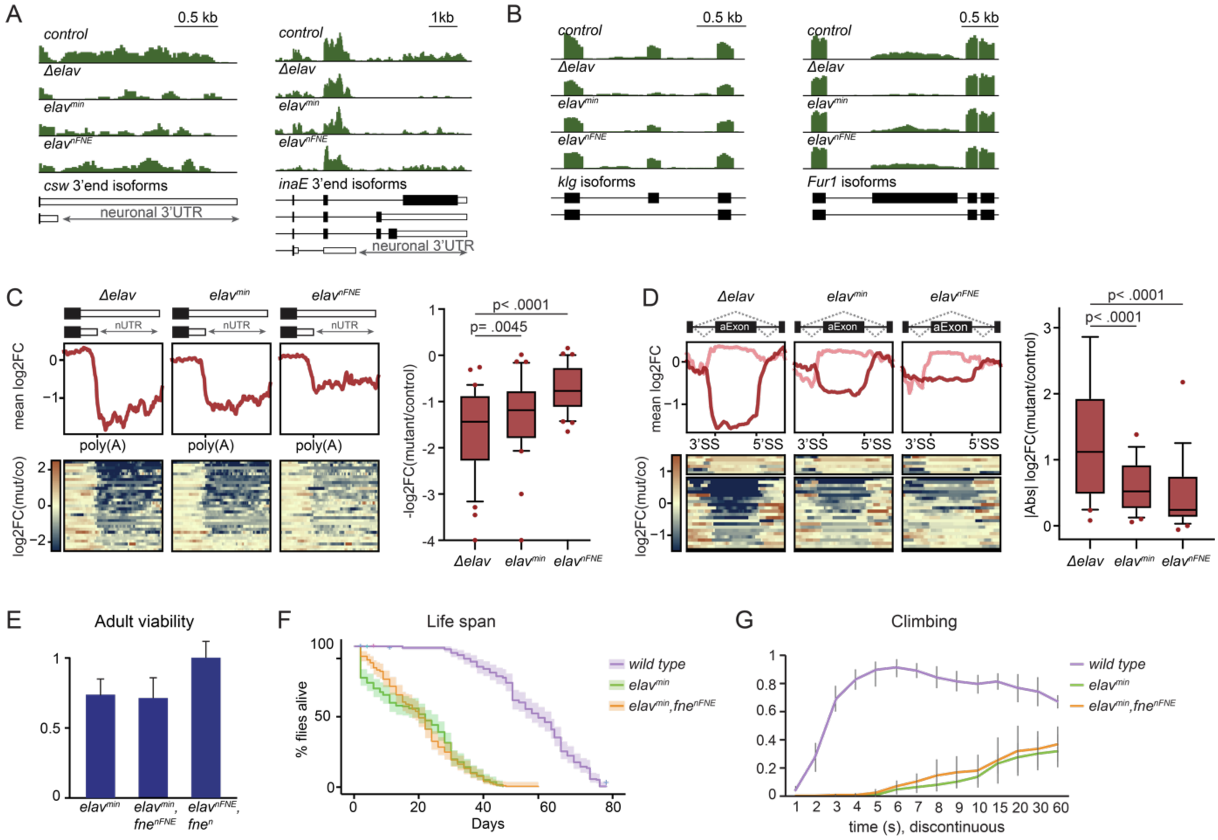

Figure S5. Early expression of nFNE rescues ELAV molecular and physiological function. Related to Figure 5.

(A, B) Representative examples of ELAV-dependent 3' UTRs (A) and exons (B) whose expression was partially (*csw*, *inaE*, *klg*) or fully (*Fur1*) restored in stage 11-12 (7-8h AEL) $elav^{min}$ embryos.

(C, D) Heatmaps and profile plots, and global quantification (box plots) of early-onset ELAV-dependent 3' UTRs (C) and exons (D), showing deregulation in *Δelav*, partial rescue of their expression in $elav^{min}$, and full rescue in $elav^{nFNE}$ embryos (stage 11-12, 7-8h AEL), compared to control (co; genotype: fne^{nFNE}). To exclude confounding effects from the endogenous *fne* gene, the genotypes *Δelav* and $elav^{min}$ also carry the fne^{nFNE} allele, which is not expressed yet at 7-8h AEL. Light red and dark red lines represent up- and down-regulated 3' UTRs and exons, respectively. Statistical significance between genotypes was calculated using Friedman's test for multiple comparisons. Profile plots, heatmaps and box plots are also shown in Fig. 4G-H, S4A-B, and 5C-F, and reproduced here for side-by-side comparison.

(E) Adult viability in flies of the indicated genotypes, measured as eclosion rate compared to expected Mendelian ratios. Eclosion rates were normalized to that of wild-type control flies. Error bars indicate mean ±SD of five biological replicates. At least 500 flies were measured for each genotype. Viability data for $elav^{min}$, $elav^{min}$,fne^{nFNE} and $elav^{nFNE}$,fne^{n} are from Fig. 1C, 3G and 5H, respectively, and reproduced here for side-by-side comparison.

(F) Life span measurement in adult flies of the indicated genotypes. The percentage of live flies is represented as a function of time after eclosion (solid line) with associated confidence intervals (shaded area). Flies scored: n=200 (wild type), n=200 (*elav*^{min}), n=200 (*elav*^{min},*fne*^{nFNE}). Data for wild type and *elav*^{min} are from Fig. 5I and reproduced here for side-by-side comparison with *elav*^{min},*fne*^{nFNE}.

(G) Measurement of climbing performance in adult flies of the indicated phenotypes, shown as the fraction of flies found above 5 cm at the indicated time points following startling. Error bars indicate mean ±SD of five biological replicates with 50 measurements each. Flies scored: n=50 (wild type), n=50 (*elav*^{min}), n=50 (*elav*^{min},*fne*^{nFNE}). Data for wild type and *elav*^{min} are from Fig. 5J and reproduced here for side-by-side comparison with *elav*^{min},*fne*^{nFNE}.

Table S2. Early-onset targets of ELAV are enriched in genes required for axonogenesis. Related to Figures 4, 5, S4 and S5.

31 APA and 29 AS early-onset ELAV targets are listed. APA type indicates whether ELAV is required for the expression of a neuronal protein-coding region (CDS) or a non-coding region (UTR). The reported function in neurogenesis is indicated for each gene based on the following Gene Ontology (GO) terms: neuronal cell adhesion (A), axon guidance (G) and nerve maturation (N).

APA targets			AS targets		
Gene	APA Type	Function	Gene	AS Type	Function
br	CDS/UTR		*Alh*	CDS	
brat	UTR		*arm*	CDS	A
Btk29A	UTR		*CadN*	CDS	A/G
CASK	UTR	N	*CG8671*	CDS	
CG12004	CDS/UTR		*chn*	CDS/UTR	
CG18265	UTR		*cic*	CDS	
CG34401	UTR		*Eps-15*	UTR	
csw	UTR		*Fak*	CDS	N
ctp	UTR	G	*Fas1*	CDS	A/G
Dscam1	UTR	G	*Fur1*	CDS	
eRF1	CDS/UTR		*klg*	CDS	A
ewg	CDS/UTR	N	*mim*	CDS	
fz2	UTR	G	*Nrg*	CDS/UTR	A/G/N
gpp	UTR		*Nrx-IV*	CDS	A/N
Hers	UTR		*osa*	CDS	
HIP-R	UTR		*Patronin*	CDS	
hth	UTR		*pod1*	CDS	G
Imp	UTR		*Ptp10D*	CDS	G
inaE	CDS/UTR		*rg*	CDS	
jing	UTR	G	*rhea*	CDS	A
Khc-73	UTR		*Sec16*	CDS	

mim	UTR		*sgg*	CDS/UTR	
nmo	UTR		*shep*	CDS	
Nrg	UTR	A/G/N	*Sin3A*	CDS/UTR	
sgg	CDS/UTR		*Slik*	CDS	
sm	UTR	G	*sqd*	CDS	
Spn	UTR		*Srrm234*	CDS	
stx	UTR		*tutl*	CDS	G
Tm1	CDS/UTR		*β-Spec*	CDS	G
Trl	UTR				
Trx-2	UTR				
